# Evaluation of potential human health risks associated with Li and their relationship with Na, K, Mg, and Ca in Romania’s nationwide drinking water

**DOI:** 10.3389/fpubh.2024.1456640

**Published:** 2024-09-23

**Authors:** Andreea Maria Iordache, Cezara Voica, Carmen Roba, Constantin Nechita

**Affiliations:** ^1^National Research and Development Institute for Cryogenics and Isotopic Technologies—ICSI, Râmnicu Vâlcea, Romania; ^2^National Institute for Research and Development of Isotopic and Molecular Technologies, Cluj-Napoca, Romania; ^3^Faculty of Environmental Science and Engineering, Babes-Bolyai University, Cluj-Napoca, Romania; ^4^Department of Biometry, National Research Institute in Forestry Marin Dracea – ICAS, Bucharest, Voluntari, Romania

**Keywords:** geochemistry, lithium, minerals, surface water, groundwater, provisional reference dose

## Abstract

**Background:**

Increasing lithium (Li) demand worldwide due to its properties and role in renewable energy will raise water reservoir pollution and side effects on human health. Divergent results regarding Li concentration in water and affective disorders are found in the literature, which is why regional reports are expected.

**Objective:**

The present study evaluated the occurrence and human health risks resulting from oral exposure, respectively, and the relationship between alkali metals (Li, Na, and K) and minerals (Mg, Ca) in balanced purified water (bottled) and spring water.

**Methods:**

The ICP-MS technique was used to measure a national database with 53 bottled and 42 spring water samples randomly selected. One-way ANOVA, Pearson correlation, and HCA analysis were applied to assess the possible relationship between metals in water. The possible side effects of Li poisoning of water resources on human health have been evaluated using the Estimated Daily Intake Index (EDI) and Total Hazard Quotient (THQ).

**Results:**

The toxic metals (As, Hg, and Pb) were measured, and the results indicate values above the detection limit of 22.3% of samples in the case of lead but not exceeding the safety limits. Depending on the water sources, such as bottled and spring water, the Li concentration varied between 0.06–1,557 and 0.09–984% μg/L. We found a strong positive correlation between Li and Na and Mg, varying between bottled and spring waters (*p*% <%0.001). Li exceeded the limit set by the Health-Based Screening Level (HBSL) in 41.37 and 19% of bottled and spring water samples. The oral reference doses (p-R_f_Ds) for the noncancer assessment of daily oral exposure effects for a human lifetime exceeded threshold values. The THQ index shows potential adverse health effects, requiring further investigations and remedial actions in 27.58% of approved bottled waters and 2.38% of spring waters.

**Conclusion:**

We can conclude that water is safe based on the Li concentration found in drinking water and supported by a gap in strict regulations regarding human Li ingestion. The present study can serve decision-makers and represent a starting database with metals of interest for further clinical studies. Decision-makers can also use it to find solutions for sustainable management of clean and safe drinking water.

## 1 Introduction

Lithium (Li) represents one of the most essential metals associated with the transition to renewable energy due to its high reactivity (1). Transactions in the Li market have increased in the last decade, mainly based on developing Li batteries for electric vehicle propulsion (2). Studies forecasting the Li trends indicate a peak in Li production at 741,000 MT in 2041, which is exceptionally high considering the low recycling percentage (3). Concerns about environmental distribution related to its recycling cycle and the extensive use of items containing Li are becoming more widespread (4). In this context, surface water is the most vulnerable since there is a lack of technologies that are capable of fully recovering Li ions from wastewater treatment plants (5). Li concentrations in natural and bottled drinking waters cover a wide range and have been reported worldwide, e.g., in the east of England (6), Chile (7, 8), Portugal (9), United States (10, 11), Hungary (12), China (13), Japan (14, 15), Nigeria (16), Bangladesh (17), France (18), Greece (19), and Romania (20, 21). The amount of Li in underground water is very high compared to surface water and needs close attention and monitoring (22). In northern Argentina, high levels of Li (1,000 μg/L) were found in drinking water, and the values were even higher in human urine (Li: 4,550 μg/L) (23).

There is an increased susceptibility to dangerous metals in the terrestrial food chain, and it has been documented that they can potentially harm human health (21, 24). Exposure to Li from natural ingestion and long-term treatment, as found in northern Argentina, where the range in drinking water varied between 8 and 1,005 μg/L, induced in women’s urinary samples a concentration of 3,910 μg/L that were associated with thyroid dysfunction (25). Still, the Li toxicity level is unknown, especially since there are no regulatory standards for Li in different environmental matrices. Li fluctuates with natural and anthropogenic activities in water, so quantification and risk assessment are essential for a sustainable society (21). The literature discusses the occurrence of Li in drinking water and its potential pharmacological and health effects (26). The therapeutic use of Li is associated with the treatment of bipolar disorder (27) and reducing suicide in patients with mood disorders (28). Different research results were found in the literature indicating that regional and local influences on human behavior are essential for quantifying the relationship between Li in drinking water and suicide rate. Those results are often divergent and depend on the region. Thus, the Li content in tap water in Japan was correlated negatively with the suicide standardized mortality ratio, indicating that an even lower concentration in water is essential for reducing suicide risk (14, 15). On the other geographic region, as in Texas, only higher Li concentrations in public drinking water were correlated with a reduced suicide rate (26, 29). Contrary to both examples previously exposed, several studies have not confirmed a positive relationship between Li in water and suicide rates, such as in the East of England in the period 2006–2008, where the relationship was insignificant (30, 31). Even so, one can discuss the subjectivism of concluding only based on drinking water and not consider a more complex dietary plan that can include other promising products of Li’s daily intake, including meat, eggs, vegetables, and fruits (21). A recent study based on the Danmark nationwide cohort indicates that Li concentration in the human body does not increase the risk of developing physical affection (32). Systematic reviews and meta-analyses on pre-clinical and clinical studies also sustain Li neuroprotective effects in Alzheimer’s and Parkinson’s affections (33, 34). The analysis of 53 Lithuanian municipalities during a two-month period using a linear model predicted that the incidence of affective disorders and Li levels in drinking water are associated with a higher ratio of attempted to complete suicide (35). Thus, questions regarding the dose effects of natural Li intake must be addressed since it is not classified as an essential trace element, even if recommended at 1 mg/day per 70 kg of body weight (36).

Other alkali metals, including sodium (Na) and potassium (K), had a significant research interest. Thus, it was observed that Na uptake from dietary sources, including drinking water, has a critical role in the human body in maintaining extracellular water balance, osmotic pressure, homeostasis, and normal neuromuscular function (37, 38). The European Commission indicates no upper threshold for Na from dietary sources, especially in regions with an excessive natural occurrence of various nutritional sources. Still, they recommend a tolerable intake level of 100 mmol (2.3 g)/day (e.g., <1,500 mg/day) (39, 40). Excessive amounts of Na contribute to cardiovascular diseases, inflammation, and obesity (41) and can raise the risk of colorectal, lung, renal, and stomach cancers (42, 43). The role of K in the human body is mainly at the intracellular osmolarity level and in maintaining acid–base equilibria (44). Hyperkalemia (>5.5 mmol/L) increases kidney diseases and diabetes (45, 46). The research results have highlighted that diets with a higher amount of K are beneficial in preventing cancer cell formation through changing hormone levels (47). The toxicity of As, Hg, and Pb is well-known, and it is worth mentioning that around 20% of human contamination originates from tap water.

Minerals at their optimum concentrations are protective against systematic disorders due to their role in structural components, biomolecules, and physiological functions (48). However, excessive nutrient assimilation represents a risk for various organ and system function failures and recently was observed to increase the incidence of cancer, possibly compounding the side effects with other toxic metals from similar sources (49, 50). Magnesium (Mg) is involved in cellular and physiological functions and processes (hypothalamus, neurotransmitters), but hypermagnesemia induces kidney failure and, in some cases, can be life-threatening (51–53). A low concentration of Mg in the human body is associated with pancreatic, prostate, colorectal, ovarian, and lung adenocarcinoma cell proliferation (54). It is still unknown if ingesting Mg in excess can be a solution to prevent the occurrence of cancer, even if this microelement can reduce the incidence (55). The recommended dietary allowance (RDA) for adults is set at 400–420 mg/day, and lower levels are associated with mood disorders (56). Calcium (Ca) is involved in multiple vital functions and disorders induced by hypercalcemia, such as dysfunctions in the endocrine system and kidney and heart diseases, mainly when it is associated with phosphorus ions (57). A high Ca ion concentration in the blood possibly protects against colorectal, breast, and prostate adenocarcinoma and is a promising option for developing future generations of anticancer drugs (58).

Even though several review studies have focused on evaluating global-scale alkali metal distributions, toxic elements, and the toxicity of macronutrient contents in drinking water, multiple terrestrial-scale geochemical data are required due to ever-increasing demand and the projected increased amount of waste. In addition, the current knowledge regarding toxicity to the human body must be continuously updated with accurate data and, most importantly, correlated with human health. An important aspect is that Li is not removed from drinking water with current treatment technologies, increasing the importance of regional monitoring studies. Thus, in the present study, we aimed to (i) build a database with metals concentrations, namely Li, As, Hg, Pb, Na, Mg, K, and Ca in drinking and spring water from Romania; (ii) analyze the occurrence and human health risks associated with Li oral ingestion via water; (iii) assess the relationship between Li and Na, Mg, K, Ca and their geospatial footprint in balanced purified water (bottled) and spring water for daily consumption in Romania; (iv) to determine the estimated daily intake index, the provisional chronic and subchronic reference dose (p-R_f_D), and the target hazard quotient (THQ) index using Li concentration in water. The hierarchical cluster analysis and correlation analysis were used to evaluate possible relationships between metals in water. The one-way ANOVA analysis was used to identify the significant differences between the chemical profiles of bottled and spring waters. The study represents a starting point for creating a regional database with Li, As, Hg, Pb, Na, Mg, K, and Ca, which can be further enriched and analyzed comparatively with chronic disease.

## 2 Materials and methods

### 2.1 Sampling and preparation

A national database that included 53 bottled and 42 spring water samples randomly selected was investigated. The bottled waters were purchased from local supermarkets and local food stores and divided into (i) mineral waters, which fall into the category of recognized waters according to national regulations, order 116/2023 (regarding the approval of the list of natural mineral waters recognized in Romania) (*n* = 29, hereafter approved waters); (ii) mineral waters that are not mentioned in legislation but are commercialized on the market (*n* = 12, hereafter unapproved); and (iii) 12 samples from foreign regions, including Hungary, Italy, Serbia, Fiji, France, Germany, Austria, and Bulgaria were used for comparison. The mineral water was bottled in polyethylene terephthalate (PET) and glass, and there was no contamination with the packaging material. The spring waters were collected from remote areas (pastures, forest) or adductions specially built to serve the local population with drinking water. In this case, the water is consumed daily without being filtered or pre-processed, contributing to metal intake from supplementary sources. Drinking water was mineralized with a 1% HNO_3_ solution. The other studied samples were subjected to microwave-assisted nitric acid digestion using a closed iPrep vessel speed system, the MARS6 CEM One Touch. The digestion vessels were cleaned with 10 mL of HNO_3_ using the microwave cleaning program and rinsed with deionized water. The water samples (45 mL + 5 mL of 69% HNO_3_) were digested according to the digestion program from US-EPA method 3015 (aqueous samples). A closed iPrep vessel speed iwaveJ system, the MARS6 CEM One Touch, was used to decompose the organic compounds in the water and extract the target elements according to the one-stage temperature-controlled digestion program, Microwave Digestion of Water (CEM Mars 6 Method Note Compendium, 2019). After complete digestion and cooling, the samples were filtered, transferred to 50 mL graduated polypropylene tubes, and diluted to volume with deionized water.

### 2.2 ICP-MS method

The inductively coupled plasma mass spectrometry technique (ICP-MS) was used to determine the Li concentrations. A Perkin Elmer ELAN DRC (e) instrument and ultra-pure deionized water (resistivity of 18 *Ω*·cm^−1^) from a Milli-Q analytical-reagent-grade water purification system (Millipore) was used. The operational conditions were optimized using a tuning solution (Elan 6100 Setup/Stab/MassCal Solution 10 μg/L for ^9^Be and ^234^Th, from Perkin Elmer) according to the following parameters: nebulizer argon flow rate: 15 L/min.; lens voltage: 7.25 V; radiofrequency power: 1,100 W; sample uptake flow rate: 1 L/min; CeO/Ce ratio of 0.028; Ba^++^/Ba ratio of 0.030. Two certificate reference materials were used for quality control: (i). a high-purity ICP multielement calibration standard of 10 μg/mL from a 29-element ICP-MS standard in a 5% HNO_3_ matrix, produced by Perkin Elmer, Life, and Analytical Sciences, Inc. Shelton, United States; (ii) an ICP mono-element certified reference material (Li) in a 2% HNO_3_ matrix from CPAchem Zaroza, Bulgary, with a concentration of 10 mg/L in a 2% HNO_3_ matrix. Each measurement was performed in triplicate, and each result was presented as the average value. Linearity was established using calibration curves, and the instrument’s sensitivity was estimated by determining the detection limits for all the elements studied. The limit of detection (LOD) and limit of quantification (LOQ) were calculated by multiplying the standard deviation of the blank sample by 3 and 10, respectively, and then dividing by the slope of the analytical curve (Table 1). The performance parameters are correlation coefficient of *r >* 0.9999, LOQ = 0.01 μg/L (Li, As, Hg, Pb), 0.5 mg/L (Na), 1.0 mg/L (Ca, Mg), 5.0 mg/L (K); respectively relative standard deviation <0.5%. The extended uncertainty declared for the analyzed metals was in the range (11–22%).

**Table 1:** The performance parameters of A Perkin Elmer ELAN DRC (e) instrument.

Element	LOD	LOQ
Na (mg/L)	0.05	0.5
Mg (mg/L)	0.1	1.0
K (mg/L)	0.5	5.0
Ca (mg/L)	0.1	1.0
Li (μg/L)	0.01	0.001
Hg (μg/L)	0.01	0.001
Pb (μg/L)	0.01	0.001
As (μg/L)	0.01	0.001

### 2.3 Health risk assessment

Risk assessment was computed by characterizing two indices: estimated daily intake (EDI) and non-carcinogenic health risk assessment. Even if the scientific literature found, in various cases, possible associations between nutrients and severe diseases, no regulations were performed for those metals until the present. Also, no exceeding limits for toxic metals were found in bottled and spring water. Thus, the analysis of health risk assessment was performed only for Li, for which regulations were exceeded according to our findings.

#### 2.3.1 Estimated daily intake of Li via water ingestion

The estimated daily intake (EDI) (μg/kg_bw_/day) of Li was calculated by multiplying the metal concentration in water (C) (μg/L) by the daily ingestion rate of water (IR) (2 L/day) and dividing it by the average body weight of an adult person (BW) (70 kg), according to *Equation 1* (59). The EDI was calculated for Li since oral reference doses (R_f_Ds) have been set by international authorities: 2 μg/kg_bw_/day (provisional subchronic and chronic R_f_D for Li) (60).

(1) $$EDI=\frac{\left(IR\times Cw\right)}{BW}$$

where IR is the daily ingestion rate of beverages (L/day); C_w_ is the average Li concentration in water (μg/L); and BW is the average body weight of 70 kg for adults. The daily ingestion rate was calculated based on the data on annual food consumption at the national level published by the National Institute of Statistics, with reports of 114.5 L/year for bottled water.

#### 2.3.2 Non-carcinogenic health risk

The non-carcinogenic health risk associated with the presence of Li in water was estimated using the method proposed by the US-EPA (59, 61, 62) based on the target hazard quotient (THQ) index and calculated using *Equation 2*:

(2) $$THQ=\frac{EF\times ED\times IR\times C}{BW\times AT\times R_{f}D}$$

where *EF* is the exposure frequency (365 days/year); *ED* is the exposure duration [75.88 years is the average lifetime for adults according to the National Institute of Statistics (NIS, 2020)]; *IR* is the average intake rate of water (2 L/person/day); *C* is the concentration of the metal in water (μg/kg); BW is the average body weight of an individual (70 kg); AT is the average exposure time (365 days/year × 75.88 years); and R_f_D is the oral reference dose (the same as the one used to calculate the EDI). For noncancer risk assessment, AT is equal to EF × ED. The THQ indicates a potential adverse health effect and requires further investigations and possible remedial actions if values are higher than 1. In the case of a THQ ≤ 1, there is no risk to human health, even for sensitive populations.

The R_f_D is used to evaluate a daily dose capable of producing health effects during a human lifetime with a recognized uncertainty. The R_f_D evaluates the most significant and sensitive lowest-observed-adverse-effect level (LOAEL) for noncancer effects and is corrected by uncertainty and modifying factors (*Equation 3*).

(3) $$R_{f}D=\frac{\mathrm{LOAEL}}{UF_{A}\times UF_{H}\times UF_{L}\times UF_{S}\times UF_{D}\times MF}$$

where UF_A_ represents uncertainty associated with using experimental findings obtained by *in vivo* animal tests for evaluating human exposure; UF_H_ is a factor that evaluates multiple variables possibly inducing variability in human resistance to stressors (age, gender, genetic adaptation, and body weight); UF_L_ is the expected ratio of LOAEL; UF_S_ is the uncertainty of estimated chronic exposure based on subchronic exposure; UF_D_ represents the uncertainty induced by the probability of association with an unequal dataset; and MF is a modifying factor that enlarges the limits of uncertainty by possible excluded unknown aspects. The provisional subchronic and chronic R_f_D for Li based on the LOAEL reference for adverse effects in several organs and systems was divided by an uncertainty of 1,000. An ingestion rate of 2.1 mg/kg/day of serum Li was established by provisional peer-reviewed toxicology values (PPRTVs) as a basis for derivation in 2008.

### 2.4 Calculations and statistical analysis

The summary statistics are presented for each category and subcategory of water, including the mean, standard error of the mean, maximum, minimum, median, and quartiles (*q1* and *q3*). The computation control used the direct weight method with degrees of freedom (DoFs), variance of the divisor of the moment, and empirical distribution with the average interpolation for the quartiles. The Shapiro–Wilk model tested the data distribution to examine how closely the samples fit into a normal distribution. This resulted in a non-normally distributed analyte metal content in the water categories, which was emphasized by significant standard deviation values. A one-way ANOVA analysis of equal variance test was conducted using the Levene method, and the results indicated significant differences among the mean Li concentrations (*p* < 0.05). The means were compared using the Bonferroni method, and the results show significant differences (*p* < 0.05). The Bonferroni method is commonly used to correct the experiment-wise error rate after using a *post hoc* procedure to correct the family-wise error rate following analysis of variance. The relationship between the Li and Na, Mg, K, and Ca contents in the water samples was tested using Pearson correlation analysis and hierarchical cluster analysis (HCA). Correlation analysis among chemical elements was evaluated to assess similar origins. The HCA aimed to group the metals and water types into classes. HCA represents a powerful method for clustering analysis in data research, aiming to identify a hierarchy of clusters. The classification was based on the similarities, correlation matrix, and indicated origins (natural and anthropogenic) of the metals.

## 3 Results and discussion

### 3.1 Li occurrence and distribution

**Figure 1:**
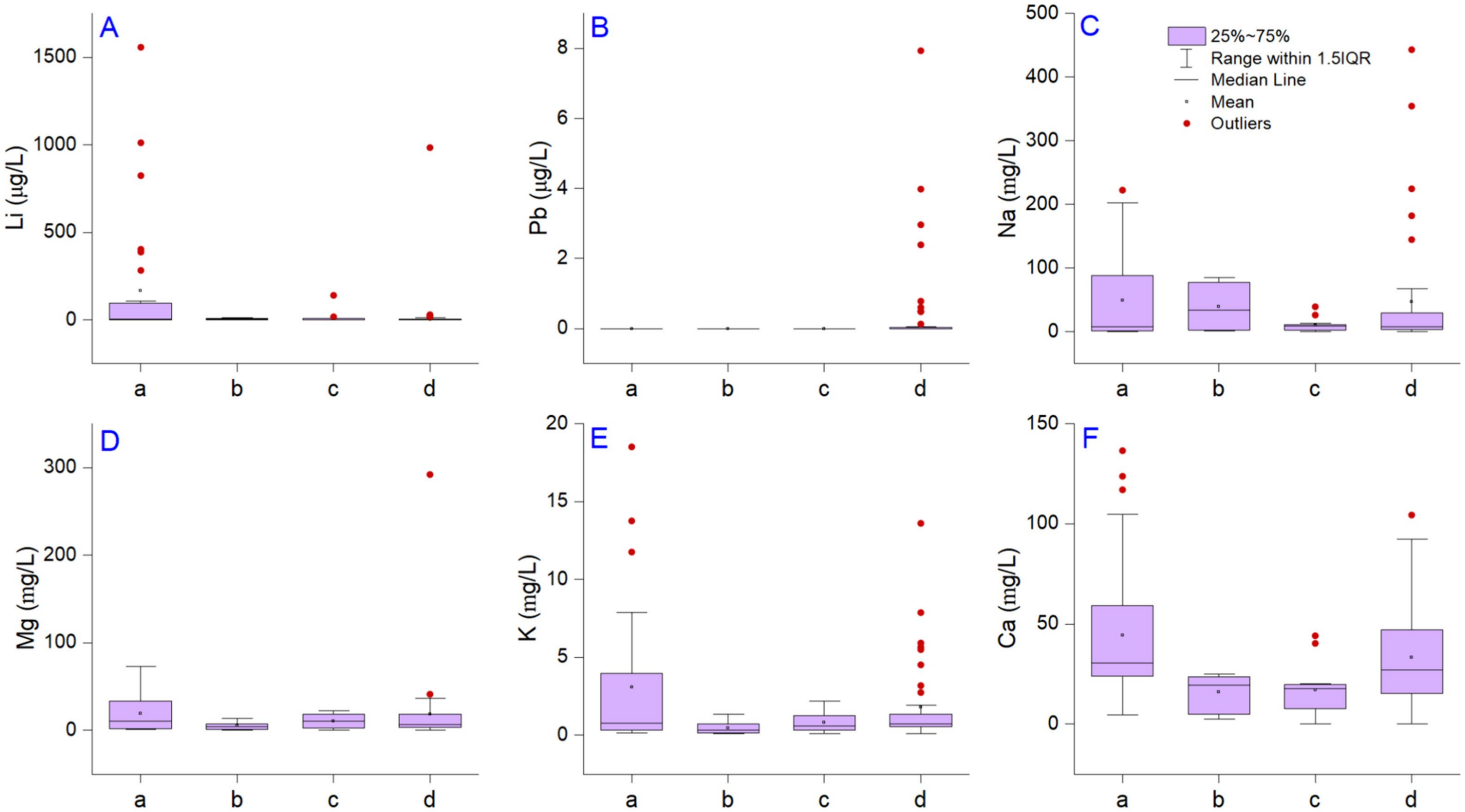
The distribution of alkali metals Li, Na, and K (panels **A, C, E**), minerals Mg, Ca (panels **D, F**), and toxic metal Pb (panel **B**). The small letters represent approved bottled waters **(A)**, unapproved bottled waters **(B)**, foreign bottled waters **(C)**, and spring waters **(D)**. The descriptive statistics and computation control was performed using the direct weight method, DF variance divisor of the moment, and empirical distribution with averaging for interpolation of quantiles.

Li is the 30th most abundant element in the continental crust and varies with lithology; it is higher in shales and granitic rocks than carbonates (63) and is frequently associated with volcanic activity, ash deposits (64–66), and saline-type deposits with LiCl reserves (67). In nature, Li in water occurs after interaction with minerals and saline lithium-bearing waters. The Li concentration in drinking water has frequently been investigated due to its pharmacological value (15, 25, 26). Our results illustrate wide analytical variability in Li content based on their water matrices, and the summary statistics are presented in Table 2. Li occurrence and distribution in commercial bottled waters (approved, unapproved, and foreign) and spring waters exceeded the detection limit in all 95 samples. The spatial distribution of the metal concentration measured in the present study correlated the largest amounts with volcanic mountain regions. A decreasing order of mean value was found as follows: approved bottled < spring < foreign bottled < unapproved bottled waters (Table 2). Based on a one-way ANOVA test, we observed no significant difference in the mean or variance between the bottled and spring waters (*F* = 0.05; *Prob > F* = 0.80). The Li measured in the bottled waters had values from 0.06 to 1,557 μg/L; in the spring water, the values varied between 0.09 and 984 μg/L (Figure 1A). Only one sample collected from central Transylvania (Cluj) in the unapproved waters was above the limit (10 μg/L). The maximum values can be associated with outliers that are unreliable in describing the dataset since they can be associated with exceptions. Thus, we used the third quartile to characterize the upper limit of Li variability in the Romanian water sample. The results show that the spring and unapproved bottled waters contain relatively similar amounts of Li (5.8–7 μg/L). When evaluating approved bottled waters, we noted that the third quartile was more than 13 times higher than other matrices. According to our findings, over 41.37% of the approved drinking waters exceeded the HBSL recommendation of 10 μg/L in Arad, Suceava, Harghita, Mures, and Covasna counties (19, 281, 822, 1,011, and 1,557 μg/L, respectively). The spring waters exceeded the HBSL recommendation by 19%, values identified in several counties, namely Salaj, Suceava, Valcea, and Covasna (10, 10, 27, and 984 μg/L, respectively).

**Table 2:** Summary statistics of alkali metals (Li, Na, and K) and minerals (Mg, Ca) in approved bottled waters (a), unapproved bottled waters (b), foreign bottled waters (c), and spring waters (d).

Element	Matrix	Mean	SE of Mean	Minimum	1^st^ Quartile	Median	3^rd^ Quartile	Maximum
Li (μg/L)	a	164	67	0.07	0.40	4.8	94	1,557
	b	3.7	1.1	0.06	0.48	2.1	7.0	10
	c	14	11	0.5	0.66	1.4	6.8	138
	d	28	23	0.09	0.71	1.7	5.8	984
Na (mg/L)	a	49	13	0.43	1.1	7.8	88	222
	b	39	11	1.2	2.4	34	77	84
	c	10	3.3	0.00	2.4	8.6	11	38
	d	46	14	0.30	3.5	7.5	29	442
Mg (mg/L)	a	19	3.9	0.80	2.2	10	33	73
	b	4.7	1.3	0.08	1.2	3.9	7.1	13
	c	10	2.4	0.00	2.4	10	18	22
	d	18	7.0	0.42	3.5	6.9	18	291
K (mg/L)	a	3.0	0.85	0.16	0.32	0.79	3.9	18
	b	0.45	0.10	0.12	0.17	0.31	0.74	1.3
	c	0.81	0.20	0.12	0.31	0.61	1.2	2.1
	d	1.7	0.40	0.10	0.53	0.73	1.3	13
Ca (mg/L)	a	44	6.5	4.7	24	30	59	136
	b	16	2.6	2.5	4.9	19	23	24
	c	17	3.9	0.04	7.8	17	19	44
	d	33	3.9	0.00	15	27	47	104

Our results are in the range of global measurements reported in the scientific literature. For example, for the inferior range of Li concentration, we can compare values with those found in the Aomori prefecture 0.0–12 μg/L (68, 69); Denmark: 0.6–30 μg/L (70); Lithuania: 0.48–35 μg/L (71); Italy: 0.11–60 μg/L (72); Greece: 0.1–121 μg/L (19); Portugal: 0–191 μg/L (73); and United States (Texas): 2.8–219 μg/L (74). The values from the upper limit measured in our study are comparable to those found in England, where the metal amount was up to 1,300 μg/L (75). Also, a Li concentration reaching 2,790 μg/L was reported in the Ogallala aquifer of northwestern Texas, where groundwater samples were collected between 2008 and 2014 from wells with depths smaller than 91.5 m (76). Thus, underground deposits highly enriched with Li can be associated with those significant values measured in drinking water. In a previous survey conducted in lowland regions from Romania, the Li concentration varied between 1.40 and 12 μg/L (20), suggesting that geological substrates are the primary regulating factor of Li′s occurrence in water. Our samples cover the entire country, including the volcanic mountains from the Eastern Carpathian. These mountains are characterized by various physicochemical compositions, e.g., carbonated, bicarbonate, sodium chloride, and iodobromated, ferruginous, arsenical, or slightly sulfurous minerals, with 3.2–22 g/L mineralization rates. This physicochemical composition is associated with a large amount of Li in water and suggests its occurrence from natural origins.

The bottled waters from foreign markets measured in the present study included samples from Bulgaria, Austria, Germany, France, Fiji, Serbia, Italy, and Hungary, and concentrations ranging between 0.50 and 138 μg/L. Based on the values previously reported in the scientific literature regarding the lowest Li concentration, we noted that the tap water derived from filtered waters is less enriched (1.5–3.6 μg/L) (12). Even so, during the present days, the reports indicated rapidly increasing Li in rivers and tap waters due to inefficient treatment protocols (77), indicating that groundwater with high total dissolved solids increased the Li concentration compared to surface water sources. Rainwater samples collected during the Summer of 1998 at Montréal Island, Canada, had even lower values (0.1–1.0 μg/L) (78). However, in this case, one can discuss the considerably raised pollution level nowadays, which contaminates the entire water cycle. In Romania, it was documented that there were high anthropic influences in industrialized areas, such as Copsa Mica. Here, Li (148 μg/L) was one of the most representative elements in groundwater associated with the battery industry (79). Leachate from waste is one of the most common sources of Li in groundwater related to anthropogenic activities (63, 80, 81). The concentration of leachate waste (batteries, ceramics, glass, lubricants, metallurgy, medicine, cosmetics, and nuclear facilities from tritium) reached 19,000 μg/L (82). A study from South Korea (Han River) demonstrated in a region with reduced Li content resulting from anthropogenic activities that the concentration downstream of Seoul (1.5 μg/L) compared to the upstream river (0.2 μg/L) resulted due to the failure of the wastewater treatment plants (77). Thus, environmental pollution with Li can be a significant issue due to inefficient recovery technologies. In bottled water, As, Hg and Pb had values below the detection limit, and only spring waters contained Pb above the detection limit in 22.3% of samples from the Bistrita and Suceava counties (Figure 1B). Lead is a harmful neurotoxin that affects multiple organs and systems, with severe implications for the normal functioning of the central nervous and brain. Pb in Bistrita samples ranged between 0.65 and 7.93 μg/L, respectively, and in Suceava, from 0.48 to 3.98 μg/L. Two protection standards set the lead poisoning level in drinking water (83, 84). Based on the World Health Organization and Environmental Protection Agency (EPA), the Lead and Copper Rule is the standard for Pb in drinking water is 10 μg/L, and the action level is regulated to 15 μg/L. Lead was below the enforcement level in all cases; no health risk assessment analysis was required. The main reason for Pb occurrence in drinking water is associated with pipes used for transport, but in our case, natural origins can be discussed (85).

### 3.2 Relationship between Li and other metals

**Figure 2:**
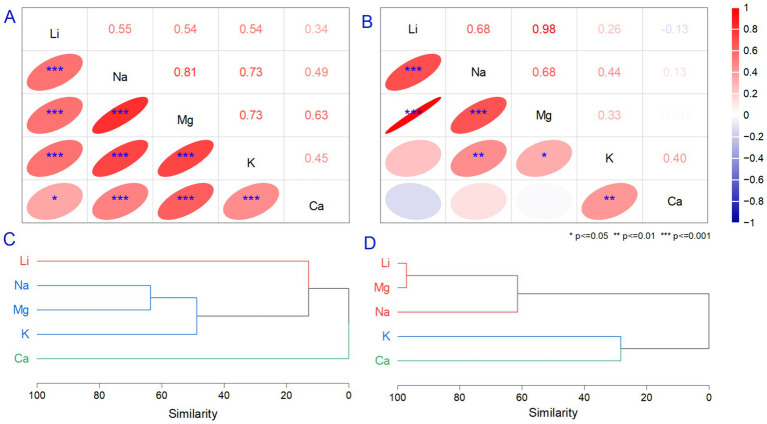
The correlation **(A,B)** and Hierarchical Cluster Analysis (HCA) dendrogram **(C,D)** are based on two datasets, which include alkali and mineral concentrations in bottled water **(A,C)** and spring waters **(B,D)**. The correlation analysis was performed for three levels of significance (*p* ≤ 0.05, *p* < 0.01, and *p* < 0.001). The HCA analysis was performed using the cluster method Ward, distance type = correlation, and clustroid found by the sum of distances.

Li in natural and bottled waters was compared to a suite of metals to understand their origins. Alkali metals naturally occur in water from geological minerals in low amounts with no environmental stress effects, except for the mineral pollucite (86). Various studies indicate different influences of alkali metals on human health, but there are no references for their relationship with macronutrients from water. The relationship between Li and Na, Mg, K, and Ca were evaluated using summary statistics correlation and HCA analysis. The range of variation for Na, Mg, K, and Ca were detailed in Table 2 and Figures 1C–F. The results show that alkali metals (Na, K) and minerals (Mg, Ca) did not exceed the recommendations. The correlation between Li and Na, Mg, and K was significant and positive (*p* < 0.001) in the case of bottled waters (Figure 2A). Still, we observed differences in the correlation coefficient when analyzing spring waters where only Li vs. Na and Mg had a significant relationship (Figure 2B), reflecting similar natural geological origins in spring waters and significant human influences on bottled waters. The correlation analysis illustrates a significant relationship between Li vs. Na, Mg, and K (*p* < 0.001) in bottled water. In the case of Li vs. Ca, the relationship was significant only for *p* < 0.05. The values were positive in all cases, representing that metal concentration has similar trends. When evaluating spring waters, we observed a strong correlation between Li and Mg (*r* = 0.98, *p* < 0.001). Similar relationships were demonstrated between Li vs. Na and Na vs. Mg (*r* = 0.68, *p* < 0.001). The positive relationship between Li and Mg can be explained by similar origins from brines such as magnesium sulfide or chloride, where both elements behave similarly in cation exchange. Previous studies that reported a relationship between Li and Na, Mg, Ca, and K in surface and groundwater found relatively similar positive correlation values. In the case of Ca and Mg, the behavior can be explained using similar behavior in removing cations and regarding Na, K, and Li acts as donors from solids, being attracted to negatively charged minerals (10). It must be mentioned that bottled drinking water is subject to national and international regulations for demineralization and desalinization. In these conditions, it can be assumed that the relationship between the Li and naturally occurring minerals differs from those in bottled waters, also observed in HCA analysis (Figures 2C,D) (87). Li and minerals are strongly associated with large concentrations of granitic pegmatites and clay mineral hectorite (88).

Minerals are abundant in the Earth’s crust, and the analyzed data appears to correlate strongly only with natural waters (unfiltered samples). Most values had a ratio between Li and other alkali metals and minerals below 1, as observed in both bottled and spring waters, and only in several spring waters exceeded this value. The ratio above 1 is associated with samples containing a very high mineral content, indicating the origins of metals from natural sources. The different chemical properties of Li can be discussed compared with other alkali metals, and they demonstrate a strong resemblance with minerals through their diagonal relationship (89). Nowadays, the Li/Na, Li/Mg, Li/K, and Li/Ca ratios can provide an archive of the previous environmental conditions, and they are used to explain past environmental processes, such as hydrothermal circulation or weathering (90–92). The compensation for Li/Na, Li/Mg, or Li/Ca carbonate in water depends on the temperature, salinity, dissolved oxygen, and pH, resulting in a new, more complex proxy for past studies (92–94). Environmental studies have used Li/Na ratios to understand the authigenic clay formation in the global Li cycle (95). Even so, the amount of minerals in bottled drinking water and spring waters can be used to assess the leaching process of the mineralogical substrate and the enrichment of natural underground and surface waters. The mineral contents are comparable with other global reports, and no exceeding limits were found in water samples.

A dendrogram (produced by HCA) integrating the alkali metals and macronutrient content in natural waters was created using the group average cluster method, correlation distance type, and *z*-score standardization to remove bias. The graphical representation of the dendrogram shows the similarities on the *y*-axis. The metals evaluated formed thre clusters of distinct groups for bottled (Figures 2A,C) and spring waters (Figures 2B,D). Li was distinctively separated from Ca, and we noted that Na, Mg, and K were associated with one group when analyzing bottled waters. In comparison, Li was associated in a group only with Mg and Na in spring waters, respectively. K and Ca formed separate groups. Ca and K were associated in a separate group in the spring water, even though their behavior varied based on their different hydration energies. Natural processes, such as the weathering of carbonate minerals, can explain the association between the macronutrients and alkali metals in the spring waters. Possible anthropogenic activities, including sewage and wastewater discharge from local industry, can also be discussed based on industrial activities. The uncertainty in the origins of Li in the water comes from the variation in their correlation similarity, which is weak. In a dendrogram produced by HCA analysis, the observation label and variables of approved, unapproved, and foreign drinking waters used for discrimination were classified into one group but connected by poor correlation similarity. We observed that the approved waters were often classified as suitable drinking water supply due to their clean appearance. Based on their high concentrations of minerals, they could be beneficial with daily ingestion.

### 3.3 Health risk assessment of Li via water ingestion

**Figure 3:**
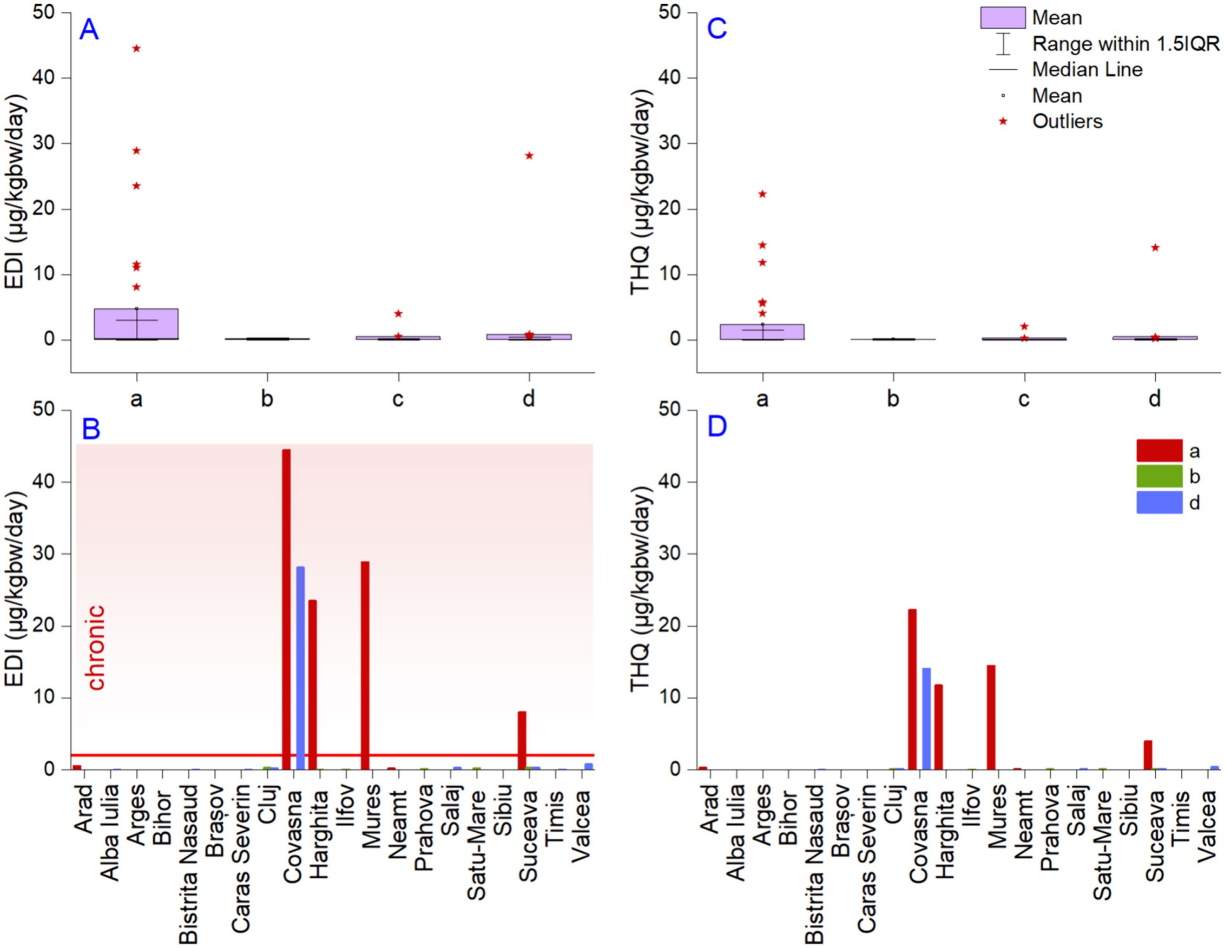
The distribution of estimated daily Li intake (μg/kgbw/day) via water ingestion **(A)** and target hazard quotient **(B)**, county maximum values; graphic representation of estimated daily intake **(C)** and target hazard quotient **(D)**. a—approved bottled waters, b—unapproved bottled waters, c—foreign bottled waters, and d—spring waters. The horizontal red line in panel **(C)** represents the limit between the subchronic and chronic R_f_D reference doses for Li.

Li is mentioned in the fifth Unregulated Contaminant Monitoring Rule due to limited exposure data in drinking water. To understand the role of the investigated metals in the human body, we calculated the estimated daily intake index (EDI) with the chronic and subchronic provisional reference dose (p-R_f_D) and target hazard quotient (THQ). According to the US Environmental Protection Agency, the daily Li intake for a 70 kg human adult ranges between 650 and 3,100 μg, corresponding to 44 μg/kg (96, 97). Recently, it was demonstrated that Li from natural waters is highly bioavailable (98); it is possible that absorption strongly relates to its accessible form in the intestinal fluid as a free ion or a complex nutrient amalgam (24). Following the daily intake methodology defined by the US-EPA (61), the maximum EDI value was found in the approved bottled waters (44 μg/kg_bw_/day), followed by the spring waters (28 μg/kg_bw_/day), foreign bottled waters (3.9 μg/kg_bw_/day), and unapproved bottled waters (0.3 μg/kg_bw_/day). Figure 3A shows several extreme values, and based on mean statistics, the values are significantly lower (4.7, 0.81, 0.42, and 0.10 μg/kg_bw_/day). In Romania, Li is not regulated, but the US Geological Survey (USGS) and US-EPA indicate a non-regulatory Health-Based Screening Level (HBSL) of 10 μg/L, which was exceeded in 41.37% of the bottled and 19% of the spring waters sampled. The excessive daily intake amounts obtained for the approved waters are not comparable with the spring waters, even though the regions are similar. One can explain elevated concentrations of Li by the interactions between water and minerals or meteoric water and the saline Li concentration. Even so, concentrations exceeding the recommended daily dose are possibly associated with chronic adverse effects in several organs and systems. Comparing our results with commercialized water from other countries also quantified in the present study, we noted that in only one sample, originating from Bulgaria, was the EDI value higher than the provisional reference dose (p-R_f_D), 3.9 μg/kg_bw_/day.

The values of the EDI exceeded the p-R_f_D for the noncancer assessment of daily oral exposure effects for a human lifetime of 2 μg/kg_bw_/day in several cases. Thus, in Covasna county, extreme values were found in the approved bottled waters (44 μg/kg_bw_/day) and spring waters (28 μg/kg_bw_/day) (Figure 3B). Exceedance of the p-R_f_D was also found in Harghita (23 μg/kg_bw_/day), Mures (28 μg/kg_bw_/day), and Suceava (8.0 μg/kg_bw_/day), but only in approved bottled waters. Thus, the local population from the above-mentioned areas could acquire more than the daily necessary dose of Li from natural waters. The minimum daily intake of Li can be obtained using unapproved waters since the maximum values were calculated for the samples collected from Cluj county. Regarding the EDI values of the spring waters, only one, originating from Covasna county, had values capable of sustaining the minimum rate of daily Li intake. The US-EPA report indicates a provisional reference dose (p-R_f_D) for the noncancer assessment of daily oral exposure effects for a human lifetime of 2 μg/kg_bw_/day.

The target hazard quotient indicates aspects similar to those of the EDI, but the amplitude of the values is lower (Figure 3C). We noted a decrease in the reported maximum THQ values in order from approved bottled waters (22 μg/kg_bw_/day) to spring (14 μg/kg_bw_/day), foreign bottled (1.9 μg/kg_bw_/day), and unapproved bottled waters (0.15 μg/kg_bw_/day). A value higher than 1 indicates potential adverse health effects and requires further investigation and possible remedial actions. In our case, 27% of the approved bottled waters and 2.3% of the spring waters exceeded this threshold. The THQ index confirmed that several samples were potentially hazardous to human health (Figure 3D). The toxicity of Li to humans has been reported to be low (99), even though various studies show that the non-supervised administration of Li carbonate in high amounts is responsible for disorders in the neuromuscular, cardiovascular, renal, and gastrointestinal systems (100). Li has been used in pharmacology for more than 50 years in the treatment of bipolar illness, and nowadays, it has disclosed underappreciated proven benefits for unipolar depression and suicide (101). Even so, various results indicate a more complicated relationship, which includes multiple variables, such as the region’s altitude, making the relationship between the Li concentration and suicide rates complex (102). Most review studies demonstrated the effect of Li on suicidal acts before the occurrence of mood stabilization, supporting the hypothesis of a possible preventative influence and not on the recurrence of disease (103).

Although very few studies have been conducted on Li and cesium’s effects on human health, it has been observed that they accumulate in the thyroid glands and present an inverse correlation at life-long exposure (104). The present study and others reinforce the importance of screening drinking water for various chemical elements to obtain further correlation and possible cumulated effects on human health due to systematic exposure (23). Groundwaters are primary drinking water sources in rural areas, and according to our results, high concentrations are expected in mountain volcanic regions. Separately for each element (Mg, Na, K, and Ca), the elevated concentrations were detrimental to human health, associated with hypertension and cardiovascular, congenital, kidney disorders, and autoimmune disorders (105, 106). Despite regulations provided for minerals in drinking water until the present based on the human capacity to assimilate extreme amounts of metals based on biological adaptation to the local environment, there was no possibility of evaluating health risk indices. Li is not the only element associated with neurological disease; Na and Ca are also involved in the mitochondrial matrix in neurons and other excitable cell functions (107, 108). Cumulating effects in the case of various concentrations can be assumed even if no specific studies are found regarding the complex interrelationship between those elements. A systematic review of suicide rates and Li content in drinking water showed reduced mortality for men compared to women, which can be explained by the Li effects of reducing impulsivity and aggression (109). Daily absorption of 93–225 mg of Li corresponds with a therapeutic response, and a serum Li concentration in the range of 500–1,200 mg/day is recommended for psychiatric treatment. A negative correlation with the Li concentration was demonstrated only in municipalities with a Li concentration above the median and a high rate of affective disorders (110). In other words, the Li in groundwater from Argentina (Andean villages), with concentrations between 8 and 1,000 μg/L, was associated with a negative impact on thyroid function (23, 25). Sparse studies confirm various clinical effects caused by significant amounts of Li ingested by humans. Generally, alkali metals can have a negative influence on human health, and contrary, the minerals are beneficial. Furthermore, high concentrations of macronutrients ingested from natural sources (food and water) are not harmful to human health since they are eliminated through urine. Na, Mg, K, and Ca have been documented to ameliorate various toxic metal-induced diseases, such as affective disorders and adenocarcinoma (55). Thus, drinking water with high amounts of minerals is recommended.

## 4 Conclusion

Our study evaluated nationwide water reserves (bottled and spring) to understand the similar origins and possible synergic effects on the public health of alkali metals (Li, Na, and K) in association with minerals (Mg, Ca). The concentration of metals varied significantly between bottled and spring waters, and a significant positive correlation between Li and Na, Mg, and K was found in bottled waters. Li correlated even stronger in spring waters, but only with Na and Mg. A secondary analysis finding added evidence on possible side effects of Li concentrations exceeding the HBSL recommendation of 10 μg/L in 41.37% of approved drinking waters and 19% of spring waters. Thus, based on human EDI and the p-RfD for the noncancer assessment of daily oral exposure, several values in bottled and spring water samples were higher than the chronic recommendation. Similar results are reflected by the THQ index, which exceeded the threshold of 1 in 27.58% of the approved bottled waters and 2.38% of the spring waters, with potential adverse health effects that require further investigation and possible remedial actions. Even so, regarding Li content in drinking water, scientific reports indicate disentangled effects on the cohort, and further studies are required to properly understand the possible effects of increased concentrations ingested by humans. The high mineral content in analyzed water can be recommended as beneficial. Our study will provide a reliable database with alkali metals and minerals, which can be further updated to evaluate various influences of metal pollution on chronic disease.

## Data Availability

The raw data supporting the conclusions of this article will be made available by the authors, without undue reservation.
